# Impact of National Economy and Policies on End-Stage Kidney Care in South Asia and Southeast Asia

**DOI:** 10.1155/2021/6665901

**Published:** 2021-05-06

**Authors:** Suceena Alexander, Sanjiv Jasuja, Maurizio Gallieni, Manisha Sahay, Devender S. Rana, Vivekanand Jha, Shalini Verma, Raja Ramachandran, Vinant Bhargava, Gaurav Sagar, Anupam Bahl, Mamun Mostafi, Jayakrishnan K Pisharam, Sydney C. W. Tang, Chakko Jacob, Atma Gunawan, Goh B. Leong, Khin T. Thwin, Rajendra K Agrawal, Kriengsak Vareesangthip, Roberto Tanchanco, Lina H. L. Choong, Chula Herath, Chih C. Lin, Nguyen T. Cuong, Ha P. Haian, Syed F Akhtar, Ali Alsahow, Mohan M. Rajapurkar, Vijay Kher, Hemant Mehta, Anil K. Bhalla, Umesh B. Khanna, Deepak S. Ray, Sonika Puri, Himanshu Jain, Aida Lydia, Tushar Vachharajani

**Affiliations:** ^1^Department of Nephrology, Christian Medical College, Vellore 632004, India; ^2^Department of Nephrology, Indraprastha Apollo Hospital, Delhi 110020, India; ^3^Department of Nephrology, “L. Sacco” Department of Biomedical and Clinical Sciences, University of Milano, Milan 20157, Italy; ^4^Department of Nephrology, Osmania General Hospital, Hyderabad 500012, India; ^5^Department of Nephrology, Sir Gangaram Hospital, Delhi 110060, India; ^6^Department of Nephrology, George Institute of Global Health, Delhi 110025, India; ^7^Clinical Research, AVATAR Foundation, New Delhi 110025, India; ^8^Department of Nephrology, PGIMER, Chandigarh 160012, India; ^9^Department of Nephrology, Armed Forces Medical College, Dhaka Cantonment, Dhaka 1206, Bangladesh; ^10^Department of Nephrology, Ministry of Health, Brunei Darussalam Medical Services, BB3910, Brunei Darussalam; ^11^Department of Nephrology, Queen Mary Hospital, Pok Fu Lam Road DD3LM 1969, Pok Fu Lam, Hong Kong; ^12^Department of Nephrology, Bangalore Baptist Hospital, Bengaluru 560024, India; ^13^Department of Nephrology, Brawijaya University, Malang 65145, Indonesia; ^14^Department of Nephrology, Serdang Hospital, Selangor 43000, Malaysia; ^15^Department of Nephrology, University of Medicine, North Okkalapa 11031, Yangon, Myanmar; ^16^Department of Nephrology, Bir Hospital, Kathmandu 44600, Nepal; ^17^Department of Nephrology, Siriraj Hospital, Mahidol University, Bangkok 10700, Thailand; ^18^Department of Nephrology, The Medical City, Pasig City 1605, Philippines; ^19^Department of Nephrology, Singapore General Hospital 169608, Singapore; ^20^Department of Nephrology, Sri Jayewardenepura General Hospital, Nugegoda 10100, Sri Lanka; ^21^Department of Nephrology, Taipei Veterans General Hospital, Taipei City 11217, Taiwan; ^22^Department of Kidney Disease and Dialysis, Vietduc University Hospital, No 40, Trangathi Street, Hanoi, Vietnam; ^23^Department of Nephrology, Viet Duc University Hospital, Hanoi 40, Vietnam; ^24^Department of Nephrology, Sindh Institute of Urology and Transplantation, Karachi 74200, Pakistan; ^25^Department of Nephrology, Jahra Hospital, Al Jahra, Kuwait; ^26^Department of Nephrology, Muljibhai Patel Urological Hospital, Nadiad 387001, India; ^27^Department of Nephrology, Medanta Hospital, Gurugram 122006, India; ^28^Department of Nephrology, Lilawati Hospital, Mumbai 400050, India; ^29^Department of Nephrology, Lancelot Kidney & GI Centre in Borivali West, Mumbai 400092, India; ^30^Department of Nephrology, Rabindranath Tagore International Institute of Cardiac Sciences, Kolkata 700026, India; ^31^Department of Nephrology, Rutgers Robert Wood Johnson Medical School, New Brunswick, NJ 08901, USA; ^32^Department of Nephrology and Hypertension, Universitas Indonesia-Dr Cipto Mangunkusumo Hospital, Salemba 10430, Jakarta, Indonesia; ^33^Department of Nephrology, Cleveland Clinic, Cleveland, OH 44195, USA

## Abstract

**Background:**

The association between economic status and kidney disease is incompletely explored even in countries with higher economy (HE); the situation is complex in lower economies (LE) of South Asia and Southeast Asia (SA and SEA).

**Methods:**

Fifteen countries of SA and SEA categorized as HE and LE, represented by the representatives of the national nephrology societies, participated in this questionnaire and interview-based assessment of the impact of economic status on renal care.

**Results:**

Average incidence and prevalence of end-stage kidney disease (ESKD) per million population (pmp) are 1.8 times and 3.3 times higher in HE. Hemodialysis is the main renal replacement therapy (RRT) (HE-68%, LE-63%). Funding of dialysis in HE is mainly by state (65%) or insurance bodies (30%); out of pocket expenses (OOPE) are high in LE (41%). Highest cost for hemodialysis is in Brunei and Singapore, and lowest in Myanmar and Nepal. Median number of dialysis machines/1000 ESKD population is 110 in HE and 53 in LE. Average number of machines/dialysis units in HE is 2.7 times higher than LE. The HE countries have 9 times more dialysis centers pmp (median HE-17, LE-02) and 16 times more nephrologist density (median HE-14.8 ppm, LE-0.94 ppm). Dialysis sessions >2/week is frequently followed in HE (84%) and <2/week in LE (64%). “On-demand” hemodialysis (<2 sessions/week) is prevalent in LE. Hemodialysis dropout rates at one year are lower in HE (12.3%; LE 53.4%), death being the major cause (HE-93.6%; LE-43.8%); renal transplants constitute 4% (Brunei) to 39% (Hong Kong) of the RRT in HE. ESKD burden is expected to increase >10% in all the HE countries except Taiwan, 10%–20% in the majority of LE countries.

**Conclusion:**

Economic disparity in SA and SEA is reflected by poor dialysis infrastructure and penetration, inadequate manpower, higher OOPE, higher dialysis dropout rates, and lesser renal transplantations in LE countries. Utility of RRT can be improved by state funding and better insurance coverage.

## 1. Introduction

The association between economic status (ES) and renal replacement therapies (RRT), dialysis infrastructure, nephrology workforce, and health policies for end-stage kidney disease (ESKD) care is not well studied in the countries within the South and Southeast Asia region (SA and SEA).

Low- and low middle-income countries (low economy; LE) are likely to foster a greater disease burden; on the contrary, they have been reporting lesser incidence and prevalence of ESKD. Due to the lack of statutory registries, this is likely a gross underestimation [1, 2]. With these limitations and existing resource crunch, the situation in LE is more complex than apparent.

Apart from the apparent impact on the disease burden, economic status directly affects therapeutic management due to the need for continuous monitoring, chronic medications, and costs towards establishment of infrastructure. With regard to renal transplants, these include maintaining transplant wait list and follow-ups, periodic viral screenings, lifelong medications, adequacy, and adherence assessments, etc. [2, 3]. Poor penetration of medical insurance, lack of government funding, limited means for out-of-pocket payment (OPP), illiteracy, lack of awareness of RRT, limited deceased donor transplant acceptance, and administrative delays contribute to inadequate delivery of ESKD care and higher mortality in these countries [4, 5].

Non-availability of representative epidemiological data of ESKD and RRT in these countries prevents substantial conclusions, effective comprehensive health policies, and outcome analyses. By comparing with the high and high middle-income countries (high economy; HE) within the SA and SEA region, we can extrapolate the likely disease burden, understand deficiencies, recommend manpower rationalization, and restructure training needs for LEs without reinventing the wheel. In this manuscript, we attempted to correlate and compare deliverables related to ESKD management and its association with national ES in SA and SEA and the impact of health policies in delivery of care.

## 2. Methods

**Figure 1:**
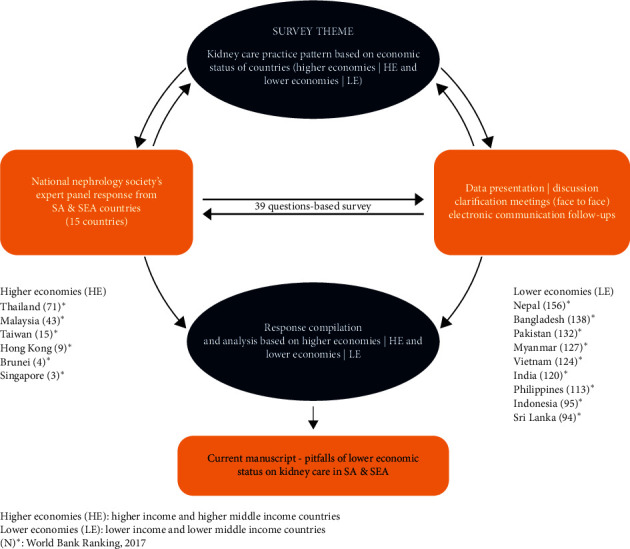
Schematic description of data collection.

An expert panel, representing national nephrology societies of SA and SEA countries, were approached with a questionnaire designed by the Association of Vascular Access and inTerventionAl Renal physicians (AVATAR, http://www.AVATAR.net.in) Foundation based in India (as per the supplementary material). The part of survey was directed towards economic status assessment and its correlation with impact on practice patterns in SA and SEA.  Figure 1 outlines the study methodology.

**Figure 2:**
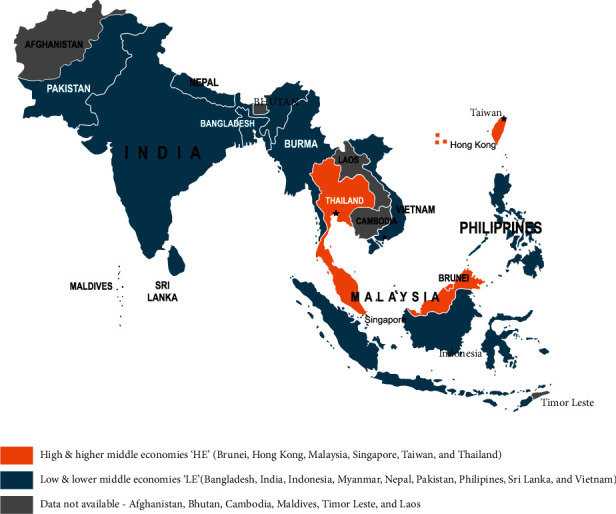
South Asia and Southeast Asia regional depiction based on economy.

An internally validated questionnaire was distributed to the national nephrology societies of all countries in the region, of which 15 countries responded, which were categorized as HE and LE, based on the economic status classification by the World Bank (Supplementary Table 1).  Figure 2 represents countries in HE that included Brunei, Hong Kong, Singapore, Malaysia, Taiwan, and Thailand while those in LE were Bangladesh, India, Indonesia, Myanmar, Nepal, Pakistan, Philippines, Sri Lanka, and Vietnam.

The responses were submitted during the 7^th^ Annual AVATAR conference held at New Delhi, India, in July 2018, by the respective society presidents/representatives based on available registries, systematic review of literature, or survey results from expert panel. The entire data was pooled and analyzed.

Continuous variables were presented as mean (±SD) and median (IQR) as appropriate. The comparison between the two groups was done using Student's *t*-test, if the variable followed normal distribution; otherwise the comparison was done using Mann–Whitney *U* test. The categorical variables were compared using Fisher's exact test. Projections for the next five years were proposed considering the growth in previous years, data provided by the representatives.

A *p*-value of 0.05 was considered as significant. Data was analyzed using SPSSv23.0.

## 3. Results

Data provided by the representatives of the national nephrology societies of 15 countries were considered for analysis.

### 3.1. Demography, Economic Status, and Healthcare Budget

SA and SEA are the most populous region worldwide with a mean population of 162.5 million. Among the two sub-groups, the mean population in HE countries (23.0 million) is ∼11 times lesser than LE (255.5 million) (Table 1).

Median per capita income (PCI) of HE (median 28192, range 5960–83250 US$) is ∼16 times higher than that of LE (median-1770, range 681–11970 US$) (Table 1). Median health expenditure as percentage of gross domestic product (% GDP) for the entire region, HE, and LE are 4 (range 3 to 7%), 5 (range 3–6%), and 4 (range 3–7%), respectively. Despite having a similar percentage GDP for healthcare, there is multifold difference in actual healthcare expenditure. As per purchasing power parity (PPP), the minimum healthcare spending in HE countries is at least 7 times higher than that in LE countries (Table 1). In summary, our study shows 11 times higher population, 16 times lesser PCI, and 7 times lower per capita median healthcare budget in LE.

The average reported ESKD incidence per million population (pmp) is 1.8 times higher in HE (305.8) than LE (167.5); and similarly, average reported ESKD prevalence pmp is 3.3 times higher in HE (1778.6) than LE (534.8) (Table 1).

### 3.2. Treatment Modality Distribution

The average distribution of hemodialysis (HD), peritoneal dialysis (PD), kidney transplant (KT), and conservative treatment (CT) in HE is 68%, 19%, 13%, and 0.2%, respectively, while in LE it is 63%, 4%, 6%, and 27%, respectively. Overall PD penetration is poor in all countries of this region except Hong Kong (46%) and Thailand (30%) (Table 2).

### 3.3. Source of Funding: Monthly Expenses towards Dialysis

Funding for dialysis by government, insurance, and OPP in HE is 65%, 30%, and 7%, respectively, while in LE it is 55%, 5%, and 41%, respectively (Table 1). Median cost of HD/month in HE and LE is US$ 586.8 and 161.8, whereas the same for PD in HE and LE is US$ 727.7 and 134.8, respectively (Table 1). Highest cost for HD (US$ 2000/month) is in Brunei and Singapore (Table 2), while the lowest cost (US$ 20–25/HD session) is in Myanmar, Nepal, Sri Lanka, and India, mainly spent as OPP. The proportion of dialysis cost in relation to annual PCI in the entire SA and SEA countries is 57%; 49% in HE and more than the 100% in LE (Tables 1 and 2).

Country-wise RRT cost and healthcare expenditure per capita in SA and SEA is given in Table 3.

### 3.4. Hemodialysis Frequencies

The average distribution of dialysis schedules in a week among HE and LE is as follows: >2 HD sessions (84% vs. 25%), 2 HD sessions (16% vs. 64%), and <2 HD sessions (0.2% vs. 11.2%). HD sessions of <2/week was 56 times more common in LE (median LE-14.7 vs. HE-0.4) (Table 2).

### 3.5. Hemodialysis Centers, Machines, and Manpower

The median number of dialysis machines/1000 ESKD population in HE is 110 (52–391) and 53 (16–90) in LE. The average number of machines/dialysis units in HE (27) is 2.7 times higher than in LE (10). The HE countries have 9 times more dialysis centers (median HE-17 vs. LE-2) and 16 times more nephrologist ppm (median HE-14.8, LE-0.94) (Table 2).

### 3.6. Dialysis Dropout Rates at One Year

The HD dropout rates at one year in HE and LE countries are 12.3% and 53.4%, respectively. Death is the major cause for dropout in HE (93.6%) and LE (43.8%). Financial constraints as a reason for dropout is ∼119 times more common in LE (mean 38.4%) than HE (0%) (Table 2). Country-wise dropout rates at 1 year are given in Table 4.

**Figure 3:**
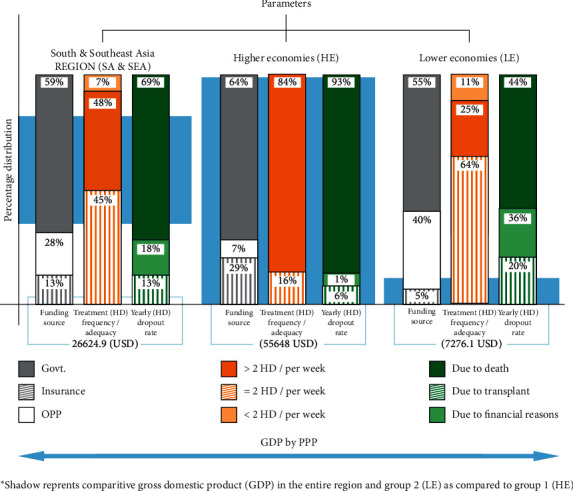
Impact of GDP on funding source, treatment adequacy, and yearly dropout.

Figure 3 depicts impact of GDP (by PPP) on funding, treatment adequacy, and dropout rate in the total region, and two economies.

### 3.7. Kidney Transplant and PD Distribution

KT in HE and LE is 13% and 6%, respectively (Table 1). The countries where KT exceeds 10% of the RRT are Singapore (18.3), Sri Lanka (21%), and Hong Kong (39%). The median penetration of PD as RRT is five times more prevalent in HE than in LE (19.1% vs. 4%) (Tables 1 and 2).

### 3.8. Projected Five-Year Growth of ESKD

ESKD burden is expected to increase by >10% in all the HE countries except Taiwan with Singapore and Brunei projecting >20% increase. Among LE countries, Sri Lanka and Nepal are expecting >20% increase in disease burden; most other nations predict an increase of 10–20%. In accordance with the ESKD burden, increment in dialysis is also expected in similar proportions (Table 4).

## 4. Discussion

### 4.1. Economic Disparity in the Region

According to a UNESCAP reports (2018) with reference to Sustainable Development Goals, SA and large part of SEA, despite representing a sizeable population, lie below the global standards [6, 7]. There is a huge variation in the population, PCI, and health expenditure between the economic groups (HE and LE) and within each group of SA and SEA region. All the representing countries within the SA are in LE category whereas there is a combination of HE and LE countries in SEA region.

Allocated healthcare budgets in most countries of SA and SEA region are 4-5% of their GDP. Due to the huge disparity in PPP between HE and LE countries, the healthcare expenditure per capita or by PPP is multifold higher in HE. It is a well known fact that financial status directly impacts the availability and affordability of better living standards and longevity.

### 4.2. Impact of Limited Resources on Growing Burden of ESKD

The median manpower (nephrologists/pmp) in HE is 15 while in LE it is 1, against the global standard of 8.83 [8]. LE countries are in a disadvantaged position due to high population, lesser PCI, and per capita expenditure on healthcare, posing serious challenges for policy makers. LE countries are also riddled with increased growth in the disease burden and this is a clarion call for addressing the fundamental issues related to delivery of kidney care in these countries.

### 4.3. Impact of Funding on RRT

Recently concluded, the International Society of Nephrology survey has shown that affordability is a barrier to the treatment accessibility apart from availability of RRT. The OPP expenses and low insurance rates were important factors affecting the access to kidney care, as well as quality of dialysis in LE and LME countries. [9].

Our study shows that, in HE, RRT is largely funded by the government or insurance bodies, which translates to prompt delivery of care and adequate logistic support with minimal financial burden on the individual or family. On the contrary, there is limited availability of state funds (55%) or insurance (5%) for RRT in LE countries, leading to a large proportion of expenses as OPP (40%) borne by the individuals or families. There is marked heterogeneity in the state funding for RRT within LE countries, with few countries (Nepal, Indonesia, Vietnam, and Philippines) covering the entire cost, others (Sri Lanka, Myanmar) providing partial cover. It is even more complex in few countries (India, Pakistan, and Bangladesh), where state funding is available only for the government employees and population below the official poverty line. We should be cognizant of the indirect costs for RRT (travel, medications, loss of livelihood due to non-availability social security funds, etc.). These in turn result in dropout from the RRT programs with time, which was highlighted by Shaikh et al. (2018) [10]; 64% of ESKD patients discontinued HD after enrolling into Rajiv Aarogyasri Community Health Insurance Scheme (RACHIS, 2008–2012) and >35% of enrolled patients discontinued their treatment within 6 months in India, emphasizing the need for overcoming challenges in indirect costs along with offering affordable dialysis for whole person care.

### 4.4. Impact of State Economy on RRT Modalities and Logistics

#### 4.4.1. Treatment Selection

Hemodialysis remains the predominant mode of RRT in SA and SEA except in a few countries with PD First policy (Thailand and Hong Kong) indicating that the choice of treatment modality is governed by state policy rather than economy. In Hong Kong, continuous ambulatory PD (CAPD) was introduced in 1980, PD First policy in 1985, and automated PD (APD) in 1989; and by 2015, ∼90% of patients were on PD (CAPD-76%, APD-14%) and only 10% on HD [11]. Similarly, the National Health Security Office of Thailand launched PD First policy under Universal Healthcare Coverage Scheme in 2008 that led to almost equal distribution of patients on HD and PD [12].

In LE countries, poor penetration and non-availability of complete data on chronic PD pose additional challenge. In India, an estimated 8500 patients are on CAPD since its inception in 1991 [13]. In Pakistan, CAPD is available since 2003, but confined to a few major cities [14]. In Bangladesh, PD started in 1986 in 10 major centers and ∼600 patients have been started on chronic PD till 2012 which has increased to 1000 in 2018. Currently, 420 patients are active on CAPD [15]. In Indonesia, Vietnam, and Myanmar, CAPD is not the preferred choice of RRT. The reasons for low PD penetration in LE countries are multifactorial and include lack of awareness, differing preferences among physicians and patients, and varying reimbursement policies for HD vs. PD by state or insurance bodies.

Though data from Singapore renal registry showed HD : PD distribution of 80 : 20 [16], it is set increase incident PD penetration to 15% in the near future. Likewise, in Brunei, HD, PD, and RT distribution was 83%, 11%, and 6%, respectively [17]. The Malaysian Dialysis and Transplant registry (2013, 22^nd^ report) recorded HD prevalence of 91% and PD prevalence of 9% among ESKD population [18]. In Taiwan registry (2012), RRT distribution was 89% of HD and 11% of PD [19].

In our study, the median prevalence trends of HD, PD, and RT in both the groups are similar but a major proportion of ESKD patients (median 7%) were on conservative treatment in LE countries, highest in Pakistan (85%) followed by Myanmar (61%), Vietnam (40%), India (27%), Bangladesh (20%), and Sri Lanka (10%). Nepal is testimony to the fact that lack of state funding is the major reason for opting conservative treatment in other LE countries, as there was a dramatic increase (∼220 times) in RRT from 0.31% of ESKD population during 1990–99 [20], to 68% in 2016 due to the state initiative of funding HD and medication costs. Similarly, Sri Lankan government sponsors 6 months of RRT to all incident ESKD patients and Myanmar state reimburses two dialysis cost per week at fixed price to the service provider (government or private) for selected ESKD patients.

In India, there is concerted government effort to set up dialysis center at all district headquarters and free treatment to the population below the official poverty line (30%). Additionally, all government employees are offered free RRT and medications. In Pakistan, charity institutions such as Sindh Institute of Urology and Transplantation run the busiest dialysis centers with 350 HD machines and over 1000 dialysis sessions/day [21]. Inevitably, LE countries have serious disparities in delivery of care to the populations living in remote areas.

#### 4.4.2. Frequency of Hemodialysis

Three times a week dialysis schedule of 4 hours each is the minimum recommended global standard for adequate HD. But in reality, our study revealed that >2 HD sessions/week was practiced by only a quarter of ESKD patients in LE and in ∼84% in HE. It was disheartening to observe that 11% ESKD population in LE countries were on <2 HD sessions/week, due to financial constraints. This phenomenon of “on-demand HD”*∗* was mostly confined to India, Bangladesh, Pakistan, and Myanmar. In Pakistan, 24% were on irregular (similar to “on-demand”) dialysis [22, 23]. In India, most of the patients were on less than twice a week schedule. Similarly, Jha [24] noticed that only affluent stayed on HD for longer periods. Inadequate dialysis sessions are a major contributor to poor one-year survival [25].

#### 4.4.3. Infrastructure

Dialysis centers and HD machines are the major components of infrastructure for the delivery of RRT. According to the available data (2016), there are 12881 dialysis machines in India, 1807 in Vietnam, 1179 in Bangladesh, 481 in Pakistan, 140 in Nepal, and 274 in Sri Lanka [26]. In LE, limited dialysis infrastructure proportionate to prevalent ESKD patients hampers the delivery of kidney care as shown in our study. This shortage was highlighted by the data published from Bangladesh in 2014 [15], where the existing infrastructure could cater to only 1/3^rd^ of prevalent ESKD patients.

#### 4.4.4. Dialysis Cost

Though the cost of dialysis (HD or PD) was more than three times higher in HE, the cumulative cost of HD exceeded the annual per capital income in LE countries, resulting in inadequate or “on-demand” HD sessions. The term “on-demand” refers to availing dialysis only when the patient is severely symptomatic or when the patient has amount to pay or rationing the funds for long-term. Variable cost of HD within LE countries is documented in the literature, lower in India [10, 27], Bangladesh [10], and at charitable or government sponsored centers in Pakistan (0–18 US$) [23, 28], but higher in Bangladesh's private setup (44–62 US$) [15, 29]. Most of the non-profitable institutions in Vietnam [30] and Myanmar [12] charge 25 US$ and 40 US$, respectively whereas in Nepal the state reimburses of 22 USD per HD session [31].

#### 4.4.5. Dropout Rate at One Year

In our study, dropout from dialysis at 1 year was 400% higher in LE, “Death alone” in HE and death and financial constraints were the reasons in LE. Early death is the reason and the consequence for high dropout rate in LE, probably due to delay in seeking medical assistance, financial constraints, and logistic reasons. In LE, financial reason for dropout is 44.5 times more common, probably contributing to inadequate HD (<2/week) (median 11.2%) and approximately one-quarter (median 27%) opting for conservative treatment option.

Financial constraints as a cause of dialysis dropout in the first 12 months were 70%, 50%, 38.4%, and 19.4% in Bangladesh, Pakistan, Sri Lanka, and India, respectively. Since RRT is completely state funded, Nepal, Myanmar, Philippines, Indonesia, and Vietnam could not provide similar data despite being LE. Change in dropout pattern with state funding was observed in Nepal and Sri Lanka, where majority of dropouts were after 6 months with the cessation of state funding. In HE countries, except Thailand (3%) and Malaysia (1%), the dropouts due to financial constraints are negligible.

#### 4.4.6. Renal Transplant

Though transplant is the best modality of RRT and economical over the long term, the number of transplants performed remained low in SA and SEA regions, especially in LE countries. Non-availability of adequate transplant centers, high cost of immunosuppression, contrary religious myths or beliefs, lack of deceased donor programs [32, 33], and lack of awareness [34] serve as a deterrent to transplants in LE countries. Scarcity of live kidney donors is an important barrier. On a brighter side, deceased donor [35], swap [36–39], and ABO incompatible transplants [40–42] are showing an increasing trend in recent years in LE. Kidney transplant, though gaining popularity, has its own roadblocks and not in the reach of many.

Majority of SA and SEA countries are LME and LE, where access to healthcare is a big challenge particularly kidney care; the region harbors a huge burden of ∼188 million patients with ESKD [43]. There is an evident mismatch between the need and supply of RRT in the LE and LME countries, resulting in inadequate patient care and increased mortality [43, 44]. Despite efforts from governments and nephrology societies to bring down the disparity in demand and supply of RRT, the situation is far from ideal. Hence, there is a need for integrated renal care approach including robust training programs for physicians, paramedics and nurses, infrastructure development, and funds to sustain [45].

Data obtained for this survey was provided by the representatives of the national nephrology societies of the participating countries of this region. As the majority of the data was based on the inputs from the registries, data extracted from the national studies, and published literature, it is assumed to be near accurate in absence of any other authentic source. In the absence of national registries in the majority of SA and SEA countries barring a few, obtaining accurate data is a big task. Despite these limitations, our study has highlighted the association between existing kidney care and economy. We may not have assessed the accurate burden, outcome including impact of comorbidities associated with ESKD. Hence, a well-designed epidemiological study covering either the entire region or country-wise including epidemiology, etiology, comorbidities treatment, and survival of ESKD patients is needed.

## 5. Conclusion

Economy has indispensable influence on the renal care in SA and SEA. The distribution of hemodialysis is similar in HE and LE, but PD and transplant are underutilized RRT modalities in both groups. Conservative ESKD management is the preferred modality only on LE because of lack of state funding, poor insurance penetration, and high out-of-pocket expenditures, while state funding and good insurance coverage are effective in enrolling majority of ESKD patients for RRT in HE. Financial constraint is a major cause for “on-demand dialysis” and increased dropout rates in LE. For adequate social security, wholesome healthcare must be included to attain sustainable development goals (SDG). State health policies and universal health coverage, government funding, efficient partnership between public and private sectors, and adequate insurance are viable solutions to improve the current state of kidney care in the SA and SEA region.

## Figures and Tables

**Table 1:** Group-wise demographics, healthcare economics, and distribution of kidney replacement therapy in SA and SEA.

Variables	Overall						Higher economies (HE)/lower economies (LE)					
	N	Mean	SD	Median	Min	Max	N	Mean	SD	Median	Min	Max
Population	15	162.5	338.8	53.7	0.43	1352.6	6/9	23/255.5	25.6/419.7	15.6/107	0.43/21.7	69.4/1352.6
PPP (GDP by PPP) (USD)	15	26624.9	30094.1	12284	2682	93905	6/9	55648/7276.1	28849.8/3431.8	56922/6775	17871/2682	93905/12811
PCI (USD)	15	16106	24290	3605	681	83250	6/9	35657.6/3071.8	29471/3450	28129 1770.3	5960/681	83250/11970
Total national health expenditure (% of GDP)	15	4.5	1.4	4.2	2.6	7.1	6/9	4.6/4.5	1.28/1.5	4.6/4	2.6/2.8	6.3/7.1
Current healthcare expenditure per capita (USD)	14	365.65	673.68	131.24	36.28	2618.71	5/9	865.21/88.12	992.72/46.09	440.83/69.29	247.04/36.28	2618.71/159.48
Current healthcare expenditure per PPP (USD)	14	811.48	1104.4	373.69	94.38	4269.96	5/9	1759.7/284.98	1477.3/132.94	1138.96/287.64	670.88/94.38	4269.96/503.5

*ESKD population*												
Incidence/million	14	226.9	100.9	190.5	100	455	6/8	305.8/167.75	102.9/45	286.8/163	171/100	455/250
Prevalence/million	12	1053	855.1	940.8	172	3219	5/7	1778.6/534.8	837.1/348.2	1306/321	1268/172	3219/1046.5

*Distribution of funding for dialysis*												
Government	14	59.6	35.1	63.3	0	100	5/9	64.8/54.6	90/51	40.7/33	0/10	100/100
Insurance	13	12.6	27.8	0.5	0	99.8	4/9	29.8/4.8	1/0.5	47.5/9.7	0/0	99.8/30
OPP	14	28.6	28.8	13.2	0	75	5/9	7.3/40.5	10/49	7.1/29.7	0/0	16.3/75

*Monthly cost (in USD)*												
HD	14	798.6	608.9	590	250	2000	5/9	1443.4/440.4	1550/400	586.8/161.8	800/250	2000/704
PD	14	757.8	592.5	550	300	2500	5/9	1301.6/455.7	1050/425	727.7/134.8	700/300	2500/706

*Distribution of RRT treatment options*												
HD (%)	15	64.9	28.1	70	10	95	6/9	67.6/63.2	27.9/29.8	78.7/67.3	15/10	87.1/95
PD (%)	15	10.1	12.2	7	0.5	46	6/9	19.1/4	15.5/3.1	10.1/3.4	8.8/0.5	46/10
KT (%)	15	9.2	10	5	0.8	39	6/9	13.4/6.4	13.7/6	7.4/5	4/0.8	39/21
CS (%)	15	16.3	26.4	0	0	84.5	6/9	0.2/27	0.4/29.9	0/20	0/0	1/84.5

GDP, gross domestic product; PPP, purchase power parity; USD, US dollars; PCI, per capita income; OPP, out-of-pocket payment; PD, peritoneal dialysis; HD, hemodialysis; KT, kidney transplant; CS, conservative.

**Table 2:** ESKD treatment demographics in SA and SEA.

Variables	Overall						Higher economies (HE)/lower economies (LE)					
	N	Mean	SD	Median	Min	Max	N	Mean	SD	Median	Min	Max
*Modality-wise distribution*												
HD (%)	15	64.9	28.1	70	10	95	6/9	67.6/63.2	78.7/67.3	27.9/29.8	15/10	87.1/95
PD (%)	15	10.1	12.2	7	0.5	46	6/9	19.1/4	10.1/3.4	15.5/3.1	8.8/0.5	46/10
KT (%)	15	9.2	10	5	0.8	39	6/9	13.4/6.4	7.4/5	13.7/6	4/0.8	39/21
Cs (%)	15	16.3	26.4	0	0	84.5	6/9	0.2/27	0/20	0.4/29.9	0/0	1/84.5

*Distribution of HD frequency*												
Less than 2 weeks	15	6.8	12.4	2	0	48	6/9	0.2/11.2	0/10	0.4/14.7	0/0	1/48
Exactly 2 weeks	15	44.9	34	50	1	92	6/9	15.9/64.2	3.9/62	25.1/24.1	1/16	65/92
More than 2 weeks	15	48.3	38.6	36	2	99	6/9	83.9/24.6	95.5/12	25/25.2	35/2	99/80

*Infrastructure and manpower*												
Absolute number of dialysis centres	14	498.9	784.5	134	6	3000	6/8	414.3/562.4	385/134	413.2/1005.1	6/48	880/3000
Dialysis centres/million population	14	8.5	9.7	2.2	0.6	27.8	6/8	17/2.1	9.5/1.5	16.8/1.8	2.1/0.6	27.8/5.5
Dialysis machines/dialysis unit	15	16.7	12.2	15	5	40	6/9	26.7/10	30/5	9.8/8.7	15/5	40/30
Nephrologists/million	15	9.2	15.3	1.72	0.04	59.8	6/9	20.8/1.5	19.6/1.9	14.8/0.94	5.6/0.04	59.8/6.5
Dialysis units/million	14	7.2	8.7	2.22	0.6	27.8	6/8	15.2/2.1	9.4/1.5	13.99/1.8	2.1/0.6	27.8/5.5

*Dropout rate*												
Yearly dropout rate	11	34.7	28.6	15.3	10	90	5/6	12.3/53.4	1.9/26.6	12/57.5	10/15	15.3/90
Due to death	10	68.7	34.1	86	15.3	99.5	5/5	93.6/43.8	7/31.8	98/43.2	88/15.3	99.5/95.5
Due to KT	10	13.3	15.8	5	0.5	46.3	5/5	5.64/20.6	5.8/19.7	2/10	0.5/4.5	12/46.3
Due to financial reasons	10	18.2	25.7	2	0	70	5/5	0.8/27.1	1.3/27.1	0/38.4	0/0	3/70

ESKD, end-stage kidney disease; PD, peritoneal dialysis; HD, hemodialysis; KT, kidney transplant; CS, conservative.

**Table 3:** Country-wise RRT cost and healthcare expenditure per capita in SA and SEA.

Countries	National population (millions)	Total national health expenditure (% of GDP)	PCI (USD)	Current healthcare expenditure per capita (USD)	Current healthcare expenditure per capita/PPP (USD)	% distribution of RRT options				Monthly cost (USD)
						PD (%)	HD (%)	KT (%)	CS (%)	HD/PD
Singapore	5.64	4.9	56,957	2618.71	4269.96	10.1	71.6	18.3	0	2000/1050
Brunei	0.43	2.6	83,250	671.40	1874.95	10	86	4	0	2000/2500
Hong Kong	7.45	5.5	32,927	440.83	841.11	46	15	39	0	DNA/DNA
Taiwan	23.73	6.3	23,330	NA	NA	8.8	87.1	4.1	<1	1550/1443
Malaysia	31.53	4.21	11,521.45	348.07	1138.96	9.5	85.7	4.7	0	867/815
Thailand	69.43	4.1	5,960	247.04	670.88	30	60	10	0	800/700
Sri Lanka	21.67	3.5	11,970	159.48	503.50	7	62	21	10	300/375
Indonesia	267.66	3	3,605	114.97	367.94	3	94	3	0	580/500
Philippines	106.65	4.5	2989	132.90	371.74	3.4	94.1	2.5	0	704/706
India	1352.62	4	2,134	69.29	253.32	0.5	67.3	5	27.2	350/500
Vietnam	95.54	7.07	1770.3	129.58	375.64	10	40	10	40	600/600
Myanmar	53.71	5.9	1,299	58.04	287.64	2	36	0.8	61.2	280/385
Pakistan	212.22	2.8	1547.9	44.59	160.56	0.5	10	5	84.5	400/310
Bangladesh	161.36	3.5	1,650	36.28	94.38	5	70	5	20	500/425
Nepal	28.09	5.8	681	47.92	150.07	5	95	0	0	250/300

RRT, kidney replacement therapy; WBR, World Bank Ranking; GDP, gross domestic product; USD, US dollars; PCI, per capita income; PD, peritoneal dialysis; HD, hemodialysis; KT, kidney transplant; CS, conservative; DNA, data not available.

**Table 4:** Country wise dropout rate & growth projections.

Countries	Total dropout rate at 1 year (%)	Dropout rate			Percentage growth projections over the next five years (2023)		
		Percentage distribution of total dropouts		
		Due to death (%)	Due to KT	Due to finances	ESKD growth	HD growth	PD growth
Singapore	11.96	99.5	0.5	0	>20	>20	10 to 20
Brunei	15.3	98	2	0	>20	>20	>20
Hong Kong	DNA	DNA	DNA	DNA	10 to 20	10 to 20	10 to 20
Taiwan	12	98.3	1.7	0	<10	<10	<10
Malaysia	12	87	12	1	>20	10 to 20	>20
Thailand	10	85	12	3	10 to 20	10 to 20	10 to 20
Sri Lanka	65	15.3	46.3	38.4	>20	>20	10 to 20
Indonesia	33.5	95.5	4.5	0	10 to 20	DNA	DNA
Philippines	DNA	DNA	DNA	DNA	10 to 20	<10	<10
India	67	43.2	37.3	19.4	10 to 20	>20	10 to 20
Vietnam	15	DNA	DNA	DNA	10 to 20	<10	<10
Myanmar	DNA	DNA	DNA	DNA	10 to 20	10 to 20	<10
Pakistan	90	45	5	50	10 to 20	<10	No growth
Bangladesh	50	20	10	70	<10	10 to 20	10 to 20
Nepal	DNA	DNA	DNA	DNA	>20	>20	>20

ESKD, end-stage kidney disease; PD, peritoneal dialysis; HD, hemodialysis; KT, kidney transplant; CS, conservative; DNA, data not available.

## Data Availability

Data are available on request to the corresponding author Dr Sanjiv Jasuja (e-mail: sanjivjasuja@yahoo.com).

## Supplementary Materials

Supplementary Materials The internally validated questionnaire drafted by AVATAR foundation's publication committee is shared to all the stakeholders including the national nephrology societies of SA and SEA to get their response on country's economic status, ESKD demographics, and ESKD practice patterns.
