# Supplementary material for: Impact of National Economy and Policies on End-Stage Kidney Care in South Asia and Southeast Asia
Source: Int J Nephrol. 2021 May 6;2021:6665901. doi: 10.1155/2021/6665901 (PMC8118744; doi:10.1155/2021/6665901)
Supplement: Supplementary Materials — The internally validated questionnaire drafted by AVATAR foundation's publication committee is shared to all the stakeholders including the national nephrology societies of SA and SEA to get their response on country's economic status, ESKD demographics, and ESKD practice patterns. [file 6665901.f1.docx]

**Current Status & Future of  Dialysis, Vascular  Access & Interventional Nephrology in South & South East Asia**

1. **Country: _____________________________________**
2. **Representative: _________________________________**
3. **Burden of ESRD**

| 4 | National population ( in millions) |  | |
| --- | --- | --- | --- |
| 5 | Incident & prevalent ESRD population  ( Per Million ) | **Incidence / million** | **Prevalence/ million** |
| 6 | %age of GDP as **Total national health expenditure** |  |  |
| 7 | Estimated Per Capita National Income  ( in USD ) |  |  |
| 8 | Top 3 Etiologies of CKD  (for e.g. DM,HT or Unknown) | **Number 1 Etiology**  **Number 2 Etiology**  **Number 3 Etiology** | |
| 9 | Average age of ESRD patient (in years)  **(at start of dialysis)** | **Male** | **female** |
| 10 | Dialysis Drop Out Rate & cause distribution at 6 months  ^(Tick applicable reason from the right Column)^ | **Rate in %** | **Death**  **Transplant**  **Financial Reasons** |
| 11 | Dialysis Drop Out rate & cause distribution at 12 months  ^(Tick applicable reason from the right Column)^ | **Rate in %** | **Death**  **Transplant**  **Financial Reasons** |
| 12 | % distribution of Patients among HD/PD/Transplant/ Conservative | **HD**  **PD**  **Transplant**  **conservative** | **( )**  **( )**  **( )**  **( )** |
| 13 | % distribution of HD frequency per week of Patients (total must be 100%) | **More than 2 per week**  **Less than 2 per week**  **2 per week** | **( )**  **( )**  **( )** |
| 14 | Total no. of nephrologists in the country  ( Number ) |  | |
| 15 | Number of dialysis centers in the country  ( Number ) |  | |
| 16 | Average number of Dialysis machines per HD unit (the commonest one) | **Less than 10**  **10-20**  **20-40**  **More than 40** | **( )**  **( )**  **( )**  **( )** |
| 17 | % distribution of Funding for dialysis (Govt,* insurance or * OPP) | **Govt**  **PPP**  **OPP** | **( )**  **( )**  **( )** |
| 18 | Are most of dialysis units supervised by Nephrologists directly? (Yes / No ) | **Yes** | **N0** |
| 19 | Monthly cost of Hemodialysis & CAPD (in USD $) |  | |

- ***PPP-PUBLIC PRIVATE PARTNERSHIP (means Government pays to private party to deliver dialysis)***
- **** OPP-OUT OF POCKET PAYMENT (means patient pays from his pocket)***

**Status Of Vascular Access**

| 20 | % of Patients undergoing “Planned initiation” of HD vs. “Unplanned initiation “ | **Planned initiation** | **Unplanned initiation** |
| --- | --- | --- | --- |
| 21 | % of **Incident Hemodialysis** patients using AVF /AVG/ * TCC/ * NON TCC | **AVF**  **AVG**  **TCC**  **nTCC** | **( )**  **( )**  **( )**  **( )** |
| 22 | % of **Prevalent Hemodialysis** Patients using AVF /AVG/ * TCC / * NON TCC | **AVF**  **AVG**  **TCC**  **nTCC** | **( )**  **( )**  **( )**  **( )** |
| 23 | Average cost of AVF/AVG /* TCC & * NTCC procedure (in USD $) | **AVF**  **AVG**  **TCC**  **nTCC** | **( )**  **( )**  **( )**  **( )** |
| 24 | AV Fistula Surgery done by: Nephrologist / Surgeons (%) | **Nephrologist** | **Surgeons** |
| 25 | AV Graft Surgery done by: Nephrologist / Surgeons (%) | **Nephrologist** | **Surgeons** |

****Tunneled Central Catheter (TCC)* * *Non Tunneled Catheter (NTCC) or Acute Catheter***

**Interventional Nephrology (IN)**

**26. Who does the following Interventional Nephrology Procedures in your country.**

| **Non Tunneled Catheter** | **(Nephrologist)** | **(Others)** | **(both)** |
| --- | --- | --- | --- |
| **Tunneled Central Catheter ( TCC )** | **(Nephrologist)** | **(Others)** | **(both)** |
| **Kidney Biopsy** | **(Nephrologist)** | **(Others)** | **(both)** |
| **AV Fistula** | **(Nephrologist)** | **(Others)** | **(both)** |
| **AV Graft** | **(Nephrologist)** | **(Others)** | **(both)** |
| **AVF Salvage Interventions** (Peripheral & Central Angioplasty) | **(Nephrologist)** | **(Others)** | **(both)** |
| **PD Catheter Placement** | **(Nephrologist)** | **(Others)** | **(both)** |

**27. What percent of nephrologists perform any IN procedure?**

|  |
| --- |

**28. The common challenges to practice of Interventional Nephrology? ^(Can check more than one box if appropriate)^**

| **Time Constraint** |  |
| --- | --- |
| **Lack of Backup Support** |  |
| **No Formal Training** |  |
| **Cost Issues** |  |
| **Fear of Medico Legal Issues** |  |
| **Lack of Incentive** |  |
| **Specialised for a Non Interventional Nephrology Practice** |  |
| **Not Applicable to our Institution / Practice** |  |

**29. Do most of the Institutes run formal training programs for following procedures in your country? ^(Check the box you feel appropriate)^**

| **Kidney Biopsy** |  |
| --- | --- |
| **Tunnelled Catheter Placement** |  |
| **Non Tunnelled Catheter Placement** |  |
| **AV Fistula Surgery** |  |
| **AV Graft Surgery** |  |
| **AV Fistula / Graft salvage procedures** |  |
| **PD Catheter Placement** |  |

**30. Are Nephrology setups in your country equipped / use / or have access to equipment like?**

| **Ultrasound Machine** | **yes** | **No** |
| --- | --- | --- |
| **Fluoroscopy / C- Arm / Cath lab** | **yes** | **No** |

**31. Does some nephrologists do cath-lab procedures in your country?**

| **Like Central or Peripheral Venous Angioplasty +/- Stenting** | **yes** | **No** |
| --- | --- | --- |
| **Renal Artery Angiography / Angioplasty / Stenting** | **yes** | **No** |

**Vascular Access Surveillance (VAS)**

**( check the box you feel appropriate )**

| **32** | **% Of Primary Failure rates of AVF and TCC (at 3months)** | **AVF Failure** | **TCC Failure** |  |
| --- | --- | --- | --- | --- |
| **33** | **Vascular Access Surveillance (VAS) Routinely Practiced In Dialysis Units** | **Yes** | **No** | **Not Sure** |
| **34** | **Who is trained as Manpower For VAS (Technicians/ Nurses/ Doctors)** | **Technicians** | **Nurses** | **Doctors** |
| **35** | **What Is The Preferred VAS Method:** | | | |
|  | **Serial Clinical Examination Of AV Access** | **Yes** | **No** | **Not Sure** |
|  | **KT/V Or URR Measurements** | **Yes** | **No** | **Not Sure** |
|  | **Routine Ultrasound Of AV Access** | **Yes** | **No** | **Not Sure** |
|  | **Any Other Technique ( name )** |  | | |

**36. Future Predictions / Directions**

| **Expected Growth Of ESKD Population in next 5 years (%)** | **No growth** | ***< 10 %*** | ***10 ~ 20%*** | ***> 20 %*** |
| --- | --- | --- | --- | --- |
| **Expected Growth Of Hemodialysis units in next 5 years (% )** | **No growth** | ***< 10 %*** | ***10 ~ 20%*** | ***> 20 %*** |
| **Expected Growth Of CAPD in next 5 years (% )** | **No growth** | ***< 10 %*** | ***10 ~ 20%*** | ***> 20 %*** |
| **Expected Quantitative Growth Of interventional Nephrology in next 5 years(%)** | **No growth** | ***< 10 %*** | ***10 ~ 20%*** | ***> 20 %*** |
| **Expected Growth Of ESKD Population in next 5 years (%)** | **No growth** | ***< 10 %*** | ***10 ~ 20%*** | ***> 20 %*** |

**37. Any Government Incentives/ Sponsored Programs/ Private Initiatives**

|  |
| --- |

**38. Challenges to Practice of HD/PD in your Country**

|  |
| --- |

**39. Expectations from & Contributions to South East Asia**

|  |
| --- |

**References Used:**
